# Perioperative Fully Closed-loop Versus Usual Care Glucose Management in Adults Undergoing Major Abdominal Surgery: A Two-centre Randomized Controlled Trial

**DOI:** 10.1097/SLA.0000000000006549

**Published:** 2024-09-30

**Authors:** Gabija Krutkyte, Arna M.C. Goerg, Christian A. Grob, Camillo D. Piazza, Eva-Dorothea Rolfes, Beat Gloor, Anna S. Wenning, Guido Beldi, Otto Kollmar, Roman Hovorka, Malgorzata E. Wilinska, David Herzig, Andreas P. Vogt, Thierry Girard, Lia Bally

**Affiliations:** * Department of Diabetes, Endocrinology, Nutritional Medicine and Metabolism, Inselspital, Bern University Hospital and University of Bern, Bern, Switzerland; † Department of Anaesthesiology and Pain Medicine, lnselspital, Bern University Hospital, University of Bern, Bern, Switzerland; ‡ Clinic for Anaesthesia, Intermediate Care, Prehospital Emergency Medicine and Pain Therapy, University Hospital Basel, Basel, Switzerland; § Department of Visceral Surgery and Medicine, Inselspital, Bern University Hospital and University of Bern; ‖ Department of Visceral Surgery, University Centre for Gastrointestinal and Liver Diseases, Basel, Switzerland; ¶ University of Cambridge Metabolic Research Laboratories, Institute of Metabolic Science, Addenbrooke's Hospital, Cambridge, UK

**Keywords:** abdominal surgery, closed-loop glucose control, diabetes technology, perioperative glycaemic control, perioperative hyperglycemia

## Abstract

**Objective:**

To assess the efficacy and safety of fully closed-loop (FCL) compared with usual care (UC) glucose control in patients experiencing major abdominal surgery-related stress hyperglycemia.

**Background:**

Major abdominal surgery-related stress and periprocedural interventions predispose to perioperative hyperglycemia, both in diabetes and non-diabetes patients. Insulin corrects hyperglycemia effectively, but its safe use remains challenging.

**Methods:**

In this two-centre randomized controlled trial, we contrasted subcutaneous FCL with UC glucose management in patients undergoing major abdominal surgery anticipated to experience prolonged hyperglycemia. FCL (CamAPS HX, Dexcom G6, mylife YpsoPump 1.5x) or UC treatment was used from hospital admission to discharge (max 20 d). Glucose control was assessed using continuous glucose monitoring (masked in the UC group). The primary outcome was the proportion of time with sensor glucose values in a target range of 5.6 to 10.0 mmol/L.

**Results:**

Thirty-seven surgical patients (54% pancreas, 22% liver, 19% upper gastrointestinal, 5% lower gastrointestinal), of whom 18 received FCL and 19 UC glucose management, were included in the analysis. The mean ± SD percentage time with sensor glucose in the target range was 80.1% ± 10.0% in the FCL and 53.7% ± 19.7% in the UC group (*P* < 0.001). Mean glucose was 7.5 ± 0.5 mmol/L in the FCL and 9.1 ± 2.4 mmol/L in the UC group (*P* = 0.015). Time in hypoglycemia (<3.0 mmol/L) was low in either group. No study-related serious adverse events occurred.

**Conclusions:**

The FCL approach resulted in significantly better glycemic control compared with UC management, without increasing the risk of hypoglycemia. Automated glucose-responsive insulin delivery is a safe and effective strategy to minimize hyperglycemia in complex surgical populations.

Major surgery induces a state of metabolic stress, mediated by inflammatory signals, counter-regulatory stress hormones, and concomitant medical procedures (eg, use of glucocorticoids, vasopressors, and nutrition support). This leads to insulin resistance, which is most pronounced on the first postoperative day and persists for 9 to 21 days after surgery.^1,2^ Consequently, at least 40% of patients undergoing major surgery are affected by intra- and postoperative hyperglycemia.^3,4^

A substantial body of literature links perioperative hyperglycemia to adverse clinical outcomes, leading to excess morbidity and mortality.^3,5–7^ Of note, patients without preexisting diabetes seem to be particularly vulnerable and were observed to have higher hyperglycemia-associated mortality compared with patients with a known diagnosis of diabetes.^8,9^ Apart from surgical stress, the use of nutrition support, particularly parenteral nutrition, is a key determinant for perioperative hyperglycemia. Up to 50% of patients receiving parenteral nutrition experience hyperglycemia, which is strongly linked to various complications (eg, infectious, cardiac, and renal complications), including higher mortality.^10^ Since postoperative nutrition support is frequent in major abdominal surgery patients and abdominal organs (pancreas, liver, gut) are critically involved in glucose regulation, this specific surgical population is highly susceptible to hyperglycemia and related complications.^6,10–15^

The most effective treatment to mitigate perioperative hyperglycemia, defined as >7.8 mmol/L and >10 mmol/L,^16^ is insulin.^17^ Beyond lowering glucose, the use of insulin helps to restore energy deficits, inhibits muscle protein catabolism, and attenuates inflammation,^18^ in line with the concept of Enhanced Recovery After Surgery.^19^ However, the use of insulin may predispose to hypoglycemia, if not properly titrated to frequently measured blood glucose levels. Due to the complexity of care and related high-time efforts,^20^ particularly in the context of global hospital staff shortages, achieving glycemic goals in the surgical population remains an unmet need.^3^

Fully closed-loop (FCL) glucose control systems, also known as artificial pancreas, offer a potential solution. These systems autonomously adjust insulin delivery through a pump based on real-time continuous glucose monitoring (CGM) and can therefore adapt to frequently changing insulin requirements,^21^ as faced in the perioperative setting. An FCL system using intravenous (IV) route for continuous glucose sensing, insulin delivery and infusion of dextrose (STG55, Nikkiso Co., Ltd), demonstrated consistent benefits in various studies in Japan involving complex surgical patients requiring postoperative intensive care.^22–24^ However, the complexity and invasiveness of the system preclude its usage over prolonged (>72 h) periods of time, such as for the stay on general wards with lower nurse-to-patient ratios, underlying the need for systems using subcutaneous (SC) route for glucose sensing and insulin administration.

The aim of this study was to investigate the efficacy and safety of FCL SC glucose control versus usual care (UC) glucose management in major abdominal surgery patients susceptible to perioperative hyperglycemia.

## METHODS

### Study Design and Participants

In this two-centre, open-label, randomized controlled trial, participants were recruited during surgical consultations before hospital admission at 2 tertiary hospitals in Switzerland: University Hospital Bern and University Hospital Basel. We included patients aged ≥18 years planned for elective abdominal surgery of ≥90 minutes duration and expected to require insulin treatment in the perioperative period (preexisting diabetes or HbA1c ≥5.7% with planned partial or total pancreatectomy), with the exception of type 1 diabetes patients. Other exclusion criteria were likely discharge within 72 hours, pregnancy, breastfeeding, any physical or psychological condition likely to interfere with the conduct of the trial or the interpretation of the results, and incapacity to give informed consent. Full inclusion and exclusion criteria are available in the Supplemental Material (Supplemental Digital Content Table S1, http://links.lww.com/SLA/F320). Eligible candidates were identified through elective surgery plans and direct referrals by local surgeons. Written informed consent was obtained from participants during the preadmission appointment, before the start of study-related procedures. The study protocol was approved by the local ethics committees (Ethics Committee Bern and Ethics Committee North-western and Central Switzerland, approval number 2022-D0034) and registered with clinicaltrials.gov (NCT 05392452). The trial was conducted in accordance with the principles of Good Clinical Practice and was overseen by a study monitor.

### Randomization and Masking

Eligible participants were randomly allocated (1:1) to either FCL or UC glucose control using a minimization method implemented through the MinimPy software.^25,26^ The factors considered in the minimization process were HbA1c (<7.5 or ≥7.5%), prestudy actual total daily insulin dose (TDD) (<30 or ≥30 units), and type of surgery (pancreatic/liver/upper gastrointestinal/lower gastrointestinal). The minimization was set up separately for each centre to ensure balance within centres. Randomization was performed within 72 hours before surgery and participants were informed about their assigned treatment modality at the time of hospital admission. The study was open-label as blinding of study treatment was not feasible. The CGM receiver in the control group was masked.

### Procedures

Participants were enrolled by clinical research fellows. At enrollment, participants’ demographics and medical history, body weight and height, nutritional status, comorbidity burden, antidiabetic medication, and details of the planned surgery (including the day of hospital admission) were assessed. A masked SC real-time CGM sensor (Dexcom G6, Dexcom) was installed up to 7 days before hospital admission on the left upper arm, unless otherwise specified by the anesthesiologists, by a clinical research fellow or a study nurse. The CGM system consisted of a small filament inserted under the skin and secured with an external adhesive patch and an attached transmitter. The sensing component measured interstitial glucose levels every 5 minutes using an amperometric glucose oxidase method and the transmitter conveyed the values to a receiver device or a mobile app. Preoperative adjustment of antidiabetic medication and nutrition support (eg, immunonutrition supplements and carbohydrate loading) was performed according to local practice. In the FCL group, UC insulin treatment (if prescribed) was withheld on the day of surgery and the participants’ CGM was unmasked and incorporated into the FCL system at the time of hospital admission. An SC cannula was inserted on the right upper arm for delivery of faster-acting insulin aspart (Fiasp, Novo Nordisk) by a study pump (mylife YpsoPump 1.5x, Ypsomed AG). The pump was controlled by the CamAPS HX app (CamDiab), which resided on an Android phone and received sensor glucose data from the Dexcom G6 transmitter. App, pump, and CGM communicated wirelessly through a low-energy Bluetooth communication protocol. The app used the Cambridge adaptive FCL model predictive control algorithm to calculate an insulin infusion rate every 8 to 12 minutes, on the basis of a compartment model of the glucoregulatory system, including submodels describing absorption of glucose in the gut and insulin in the SC tissue.^27^ In times of sensor glucose unavailability, the CamAPS HX FCL system is designed to continue to automatically adapt insulin delivery (ie, auto-mode), if capillary blood glucose values are entered hourly. Sensor glucose and insulin data were automatically uploaded to the Diasend/Glooko data management platform (https://diasend.com; https://glooko.com/) allowing for remote monitoring. The control algorithm was initialized using the participant’s body weight and estimated TDD. The nominal glucose target was set to the default of 5.8 mmol/L or 7.0 mmol/L, based on individual circumstances (nutrition type, risk for hypoglycemia, and previous glycemic control). The low glucose alert was used as per the default setting (3.1 mmol/L). FCL insulin delivery was stopped at discharge or after a maximum of 20 days after surgery. A flat basal rate profile at 20% of the estimated TDD was preprogrammed to ensure continued insulin delivery when auto-mode was inactive.

In the UC group, glucose management was at the discretion of the involved care team and was not specified by the study protocol. The standard of care differed according to the stage and setting of perioperative care and individual factors. As per routine, glucose levels during surgery and intensive and intermediate care, were controlled using IV insulin according to standardized infusion protocols aiming to keep glucose levels between 8.0 mmol/L and 10.0 mmol/L. The same protocols were accessible to the general wards, where the use of IV insulin was decided on an individual basis. SC insulin therapy on the general wards was prescribed using electronic health record-integrated insulin order sets, considering blood glucose levels and estimated insulin sensitivity. A specialist diabetes outreach team was available on both sites on demand.

At the time of discharge, all participants were fitted with a masked CGM to monitor glucose control in the postdischarge period (up to 20 days). In both study groups, antidiabetic medication at discharge was managed by the clinical care team, which was provided with the hospital CGM record to allow for treatment optimization, where appropriate.

### Outcomes

The primary outcome was the proportion of time the sensor glucose concentration was in the target glucose range of 5.6 to 10.0 mmol/L (set in line with recommendations for less stringent glucose control in this population^16^), from hospital admission to discharge or a maximum of 20 days after surgery. A single value was calculated for each subject for each treatment arm by pooling all CGM readings for the defined time period; no interpolation was performed. Secondary outcomes comprised time with sensor glucose below the target range (<5.6 mmol/L), proportion of time in hyperglycemia (>10.0 mmol/L and >20.0 mmol/L), and hypoglycemia (<3.9 mmol/L and <3.0 mmol/L), as well as mean sensor glucose and glucose variability (defined as SD and coefficient of variation of sensor glucose). Other outcomes of interest were received insulin dose, use of other medication with impact on glucose control (eg, glucocorticoids and somatostatin analogs), and nutrition support (eg, parenteral and/or enteral nutrition). Information on preadmission and postdischarge glucose control (as assessed by masked CGM), patient comorbidity, and hospitalization metrics, were collected for descriptive purposes.

Safety outcomes included hypoglycemic events with plasma glucose levels <2.2 mmol/L and clinically significant hyperglycemia (plasma glucose>20.0 mmol/L) with ketonemia (beta-hydroxybutyrate >1.0 mmol/L), as well as other (serious) adverse events related to the study procedures.

### Sample Size Calculation

The sample size was calculated to ensure a power of 90% to detect a clinically significant between-group difference of 20 percentage points in the primary outcome. The calculation was based on the assumption of a within-group SD of 16.3% and using a 2-sided *t* test at an alpha level of 0.05. The SD was estimated based on the results of a previous trial conducted in a similar setting.^28^ This resulted in a target sample size of 30 participants. Accounting for a drop-out rate of 10% and 10% of participants with <48 hours of data, we aimed to enroll 38 participants (n = 19 in each group).

### Statistical Analysis

We analyzed efficacy and safety outcomes according to the modified intention-to-treat principle. The unpaired Welch`s *t* test was used to compare means of variables conforming to normality assumptions, while the Wilcoxon rank-sum test was used otherwise. Normality was assessed using the Shapiro-Wilk test. Assumptions were inspected using graphical methods to ensure the appropriateness of the statistical methods. The proportion of time with sensor glucose in the target range (primary outcome) was compared using the unpaired Welch`s *t* test. Results for all prespecified outcomes are reported as either the mean difference between the interventions along with 95% CIs or the median of the differences corresponding to the Hodges-Lehmann estimate and its 95% CI in the case of nonparametric testing. We report values as mean ± SD or median (25th percentile, 75th percentile), unless stated otherwise. Reported *P* values correspond to 2-tailed tests, and *P* values <0.05 were considered statistically significant. Calculations of all outcomes and statistical analyses were done in *R* (version 4.0.2).

## RESULTS

A total of 70 patients were approached for enrollment at University Hospital Bern and Basel between August 2022 and October 2023, of whom 38 were eligible and provided written informed consent. Eighteen patients were randomly assigned to the FCL group and 20 to the UC group. One participant of the UC group withdrew from the study due to feeling overwhelmed by the medical diagnosis and complicated surgery planning. Due to withdrawal before surgery, data from this subject were not included in the main analysis. The CONSORT flow diagram^29^ and checklist are shown in the Supplemental Material (Supplemental Digital Content Fig. S1, http://links.lww.com/SLA/F320, Supplemental Digital Content Table S2, http://links.lww.com/SLA/F320).

The baseline characteristics of the FCL and UC groups are shown in Table 1. The population consisted of elderly, mostly male (73%) patients. In both groups, pancreatic surgery was the predominant type of surgery (55.6% and 52.6% in FCL and UC, respectively). Of the total, 16.7% in the FCL and 21.1% in the UC group had no previous history of diabetes.

**TABLE 1:** Baseline Characteristics

	FCL (n = 18)	UC (n = 19)
Age (yr)	68.2±10.9	63.9±13.0
Sex (F)	5 (27.0)	5 (26.3)
BMI (kg/m²)	29.9±6.2	25.9±4.6
HbA1c (%)	7.3±0.9	6.6±0.9
Charlson Comorbidity Index	7.7±3.1	5.9±3.1
ACS NSQIP surgical risk score	37.4±13.3	29.3±9.1
No. of NRS ≥3	13 (72.2)	12 (63.2)
No. of clinical frailty score ≥3	15 (83.3)	13 (68.4)
History of diabetes	15 (83.3)	15 (78.9)
Duration of diabetes (yr)	3.0 (0.74; 21.3)	8.4 (13.0; 31.5)
Glucose lowering therapies at enrollment (no. of patients)		
Basal-only insulin	6 (33.3)	2 (10.5)
Basal-bolus insulin	5 (27.8)	4 (21.1)
Insulin-naïve	7 (38.9)	13 (68.4)
SGLT-2 inhibitor	8 (44.4)	4 (21.1)
Metformin	6 (33.3)	9 (47.4)
DPP-4 inhibitor	1 (5.6)	1 (5.3)
GLP-1 RA	1 (5.6)	1 (5.3)
Sulfonylurea	1 (5.6)	1 (5.3)
Insulin total daily dose at baseline (IU)*	20.0 (15.0; 28.5)	26.0 (13.0; 31.5)
Type of major abdominal surgery (no. of patients)		
Pancreatic	10 (55.6)	10 (52.6)
Upper GI	4 (22.2)	3 (15.8)
Lower GI	0	2 (10.5)
Liver	4 (22.2)	4 (21.1)

Data are mean ± SD, median (25th; 75th percentile), or n (%) unless otherwise specified.

* Only patients with insulin therapy were considered.

ACS NSQIP indicates American College of Surgeons National Surgical Quality Improvement Program; BMI, body mass index; DPP-4, dipeptidyl peptidase-4; FCL,fully closed-loop; GI, gastrointestinal; GLP-1 RA, glucagon-like peptide 1 receptor-agonist; IU, international units; NRS, nutritional risk score; SGLT, sodium-glucose co-transporter; UC, usual care.

### Glucose Management Outcomes

**FIGURE 1:**
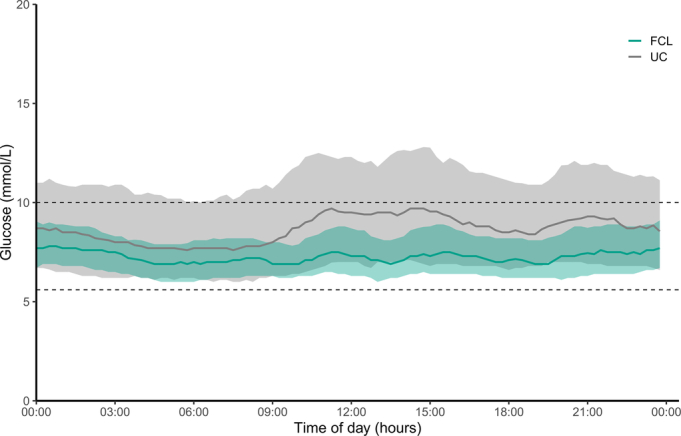
Ambulatory glucose profile. Twenty-four-hour representation during FCL (green) and UC (grey) interventions (lines indicate median, shaded areas indicate IQRs). The glucose target range (dashed lines) is 5.6 to 10.0 mmol/L (100–180 mg/dL). IQR indicates interquartile range. FCL indicates fully closed-loop; UC, usual care.

**FIGURE 2:**
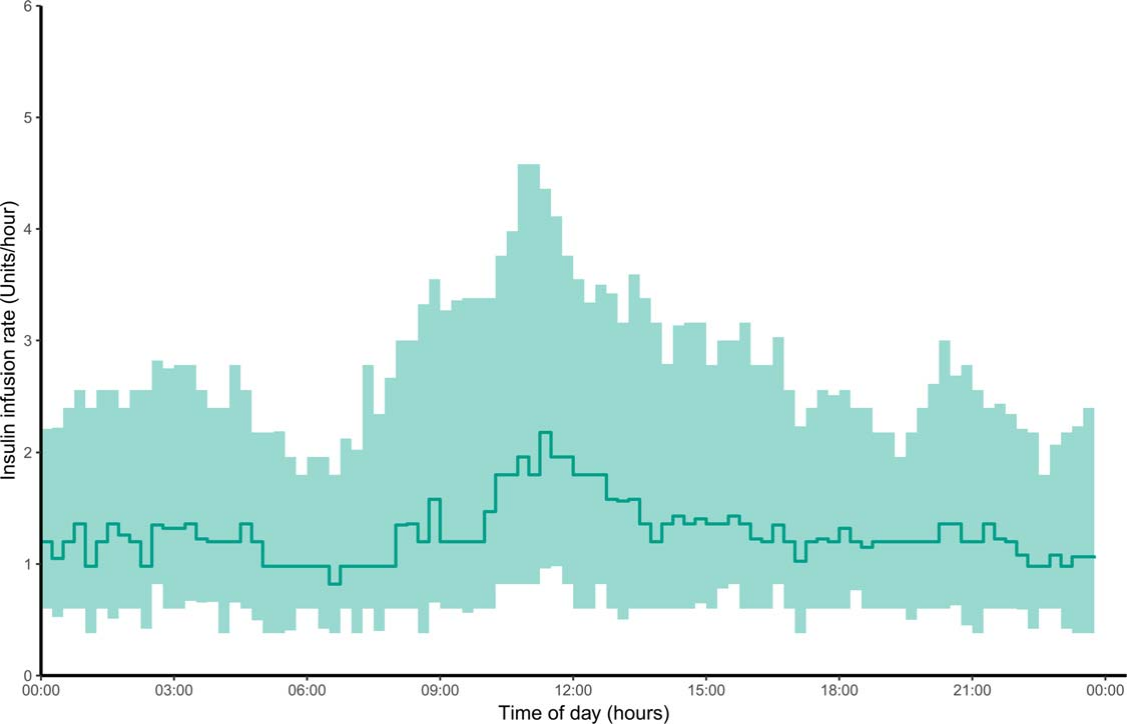
Twenty-four-hour insulin delivery profile. Twenty-four-hour representation of algorithm-directed insulin delivery during the FCL intervention (line indicates median, shaded area indicates IQR). FCL indicates fully closed-loop; IQR, interquartile range.

**FIGURE 3:**
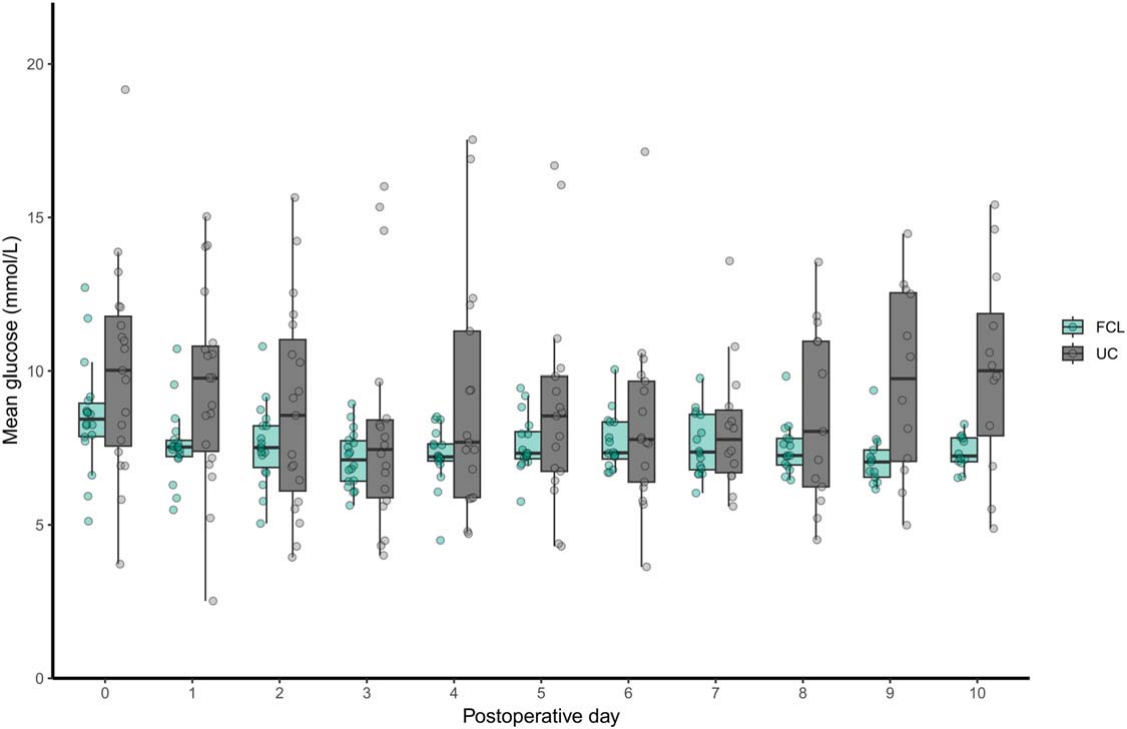
Mean daily sensor glucose values during postoperative days 0 to 10. Boxplots show median (solid line), IQR (box outline), and spread of data points without outliers (whiskers). Outliers are defined as measurements beyond 1.5*IQR (symbols). FCL indicates fully closed-loop; IQR, interquartile range; UC, usual care.

**FIGURE 4:**
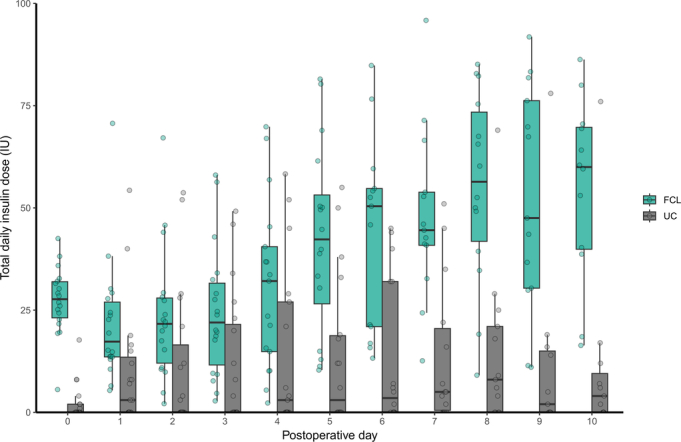
Mean daily insulin dose during postoperative days 0 to 10. Boxplots show median values (solid line), IQR (box outline), and spread of data points without outliers (whiskers). Outliers are defined as measurements beyond 1.5*IQR (symbols). FCL indicates fully closed-loop; IQR, interquartile range; UC, usual care.

Glucose and insulin metrics during the intervention period are depicted in Table 2. The proportion of time with sensor glucose levels in the target range (5.6–10.0 mmol/L) was 26.4 percentage points (95% CI: 16.0; 37.0) higher in the FCL group than in the UC group (*P* < 0.001). Mean sensor glucose concentration was significantly lower in the FCL than in the UC group (7.5 ± 0.5 vs 9.1 ± 2.4 mmol/L, *P* = 0.015). Glycemic variability during closed-loop therapy, as measured by CV and SD of sensor glucose concentration, was significantly lower compared with UC insulin therapy. The proportion of time spent with sensor glucose concentrations above the target range (>10.0 mmol/L) was significantly lower in the FCL compared with the UC group (9.1 ± 6.2% vs 31.9 ± 25.3%, *P* = 0.001), but the time spent below the target range (<5.6 mmol/L) did not significantly differ between groups. The proportion of time spent with concentrations <3.9 mmol/L and <3.0 mmol/L were low and did not significantly differ between them. Total daily insulin dose was significantly higher in the FCL compared with the UC group [34.3 IU (25.8; 44.1) vs 3.5 IU (1.1; 15.3), *P* < 0.001]. The aggregated 24-hour median interquartile range sensor glucose profile of both study treatment groups and the 24-hour median interquartile range insulin delivery profile in the FCL group are illustrated in Figure 1 and Figure 2, respectively. The evolvement of mean daily sensor glucose values and mean daily insulin dose over hospital stay are depicted in Figure 3 and Figure 4. Typical high variability of day-to-day insulin requirements is showcased in Supplemental Figure S2 (Supplemental Digital Content Figure S2, http://links.lww.com/SLA/F320), which illustrates the perioperative evolvement of FCL insulin delivery in a patient undergoing total pancreatectomy.

**TABLE 2:** Glucose Management Outcomes

	FCL (n = 18)	UC (n = 19)	*P*	Group difference (FCL - UC)	95% CI for the between-group difference
Proportion of time spent at glucose concentration; mmol/L (%)					
5.6-10.0^*^	80.1±10.0	53.7±19.7	<0.001	26.4	16.0; 37.0
>10.0	9.1±6.2	31.9±25.3	0.001	−22.8	−35.3; −10.3
>20.0	0.0 (0.0; 0.0)	0.0 (0.0; 1.8)	0.046^†^	−3.5*10^-05^	−0.2; 0.0
<5.6	9.0 (5.6; 12.9)	4.4 (1.3; 23.6)	0.35	2.7	−7.6; 6.7
<3.9	0.5 (0.0; 1.7)	0.2 (0.0; 1.9)	0.91	0.0	−0.4; 0.6
<3.0	0.0 (0.0; 0.3)	0.0 (0.0; 0.2)	0.68	0.0	−0.1; 0.1
Mean glucose concentration (mmol/L)	7.5±0.5	9.1±2.4	0.015	−1.6	−2.6; −0.3
SD of glucose concentration (mmol/L)	1.8±0.5	2.7±1.0	0.004	−0.9	−1.4; −0.3
CV of glucose concentration (%)	24.1±5.7	29.5±9.5	0.041	−5.4	−10.6; −0.2
Total daily insulin dose (IU/24 h)	34.3 (25.8; 44.1)	3.5 (1.1; 15.3)	<0.001	26.9	19.9; 33.6

^*^ Primary outcome.

^†^ The distributions of the variable differ between FCL and UC.

Data are mean ± SD or median (25th; 75th percentile). *P* values were computed using the Welch`s *T* test or Wilcoxon rank-sum test. 95% CI for the difference in the location parameters (difference in means or Hodges-Lehman estimator).

The *P* value, calculated using the Wilcoxon rank-sum test, represents the probability that a randomly selected value from FCL is greater than a randomly selected value from UC.

CV indicates coefficient of variation; FCL, fully closed-loop; IU, international units; UC, usual care.

### Other Metrics of Perioperative Care

Other metrics of perioperative care are depicted in Table 3. The mean duration of surgery was 5.4 ± 4.6 and 4.7 ± 2.7 hours in the FCL and UC group, respectively. Postoperatively, 61.1% of FCL patients received parenteral nutrition support, of which 16.7% were in combination with enteral nutrition. In the UC group, 26.3% of patients received parenteral nutrition (of which 5.3% were in combination with enteral nutrition). The length of hospital stay was 15.0 days (9.3; 25.8) and 14.0 days (7.5; 15.0) in the FCL and the UC group, respectively. Postoperative complications, as assessed by the Comprehensive Complication Index (composite score calculated as a sum of all complications up to 30 days after surgery that are weighted for their severity)^30^ are reported in Table 3, with Clavien-Dindo grading in Supplemental Table S3 (Supplemental Digital Content Table S3, http://links.lww.com/SLA/F320).

**TABLE 3:** Characteristics of Perioperative Care

	FCL (n = 18)	UC (n = 19)
Surgery duration (h)	5.4±4.6	4.7±2.7
No. of receiving preoperative carbohydrate loading	4 (22.2)	6 (31.6)
No. of receiving preoperative immunonutrition supplements	3 (16.7)	1 (5.3)
No. of receiving intraoperative glucocorticoids	9 (50.0)	13 (68.4)
No. of receiving somatostatin analogs	10 (55.6)	4 (21.1)
No. of receiving parenteral nutrition	11 (61.1)	5 (26.3)
of which combined with enteral nutrition	3 (16.7)	1 (5.3)
No. of receiving enteral nutrition	2 (11.1)	4 (21.1)
No. of receiving postoperative non-insulin glucose-lowering agents*	7 (38.9)	6 (31.6)
No. of planned stay at the IMC/ICU	17 (94.4)	13 (68.4)
No. of unplanned stay at the IMC/ICU	3 (16.7)	3 (15.8)
No. of receiving postoperative glucocorticoids	3 (16.7)	1 (5.3)
Length of stay (d)	15 (9.3; 25.8)	14 (7.5; 15.0)
Comprehensive Complications Index	35.9±26.2	18.5±17.1
No. of receiving postdischarge glucose-lowering agents	16 (100)†	12 (63.2)
Basal insulin	14 (87.5)	8 (42.1)
Bolus insulin	10 (62.5)	7 (36.8)
SGLT-2 inhibitor	6 (37.5)	6 (31.6)
Metformin	5 (31.3)	7 (36.8)
DPP-4 inhibitor	2 (12.5)	0
GLP-1 RA	1 (6.3)	2 (10.5)
Sulfonylurea	0	1 (5.3)

Data are mean ± SD, median (25th; 75th percentile), or n (%).

* Includes SGLT-2 inhibitors, metformin, sulfonylurea, GLP-1 RA, DPP-4 inhibitors.

† Patients deceased during hospitalization not considered.

DPP-4 indicates dipeptidyl peptidase-4; FCL, fully closed-loop; GLP-1 RA, glucagon-like peptide 1 receptor-agonist; ICU, intensive care unit; IMC, intermediate care; SGLT, sodium-glucose co-transporter; UC, usual care.

### Preadmission and Postdischarge CGM Metrics

Masked CGM recording before hospital admission was performed for 3.1 ± 2.2 days in the FCL group and 3.8 ± 1.8 days in the UC group (Supplemental Material, Supplemental Digital Content Table S4, http://links.lww.com/SLA/F320). The mean sensor glucose concentration was 11.5 ± 4.5 mmol/L in the FCL group and 10.4 ± 3.0 mmol/L in the UC group. The proportion of time with sensor glucose value between 3.9 mmol/L and 10.0 mmol/L, as recommended for the outpatient setting,^31^ was 53.0% ± 39.9% for the FCL group and 54.4% ± 32.3% in the UC group.

In the postdischarge period, the mean glucose concentration was 9.1 ± 1.5 mmol/L in the FCL and 9.3 ± 2.2 mmol/L in the UC group. Time with hypoglycemic values was negligible in both groups, before and after hospitalization. Postdischarge glucose-lowering medication is depicted in Table 3.

### Utility Metrics, Safety, and Device Issues

During the study treatment period, sensor glucose data were available for 99.1% (98.3; 99.4) of the time in the FCL group, and 97.6% (94.3; 98.7) in the UC group. FCL was operational for 99.8% (98.8; 100.0) of the study treatment period and for 99.8% (99.4; 100.0) of the time when sensor glucose data were available.

FCL glucose control was temporarily suspended in one participant due to a magnetic resonance imaging scan (bridged with 5 units of insulin detemir). One FCL patient had to prematurely stop study participation after 4 days of therapy at the discretion of the clinical team, due to acute liver failure requiring critical care. No episodes of severe hypoglycemia, significant hyperglycemia with ketonemia, or any study-related serious adverse event occurred in either group. Four serious adverse events occurred in 3 FCL subjects [2 portal vein thrombosis requiring revision surgery and 2 deaths (one due to multiple organ failure and one due to hemorrhagic shock)], none of them study-related.

A total of 26 device deficiencies occurred in both groups, consisting of 18 CGM sensor error alerts (of which 15 required sensor replacements), 5 pump occlusion alarms requiring infusion set replacements, and 3 pump electronic errors in 3 different pumps.

## DISCUSSION

In this two-centre randomized controlled trial, we compared the efficacy and safety of FCL SC glucose control with UC glucose management in patients undergoing major abdominal surgery and prone to develop perioperative hyperglycemia, from hospital admission to discharge or a maximum of 20 days. The FCL approach resulted in significantly better glucose control by increasing time spent in the target range, lowering mean sensor glucose and glycemic variability, while not increasing the risk of hypoglycemia.

The mean glucose concentration in the UC group was 9.1 ± 2.4 mmol/L (or 1.6 mmol/L higher than in the FCL), a glucose concentration range that was clearly linked with postoperative morbidity and mortality in several large-scale observational studies.^3,7,14,32,33^ The dose-response relationship between the degree of hyperglycemia and complications was particularly evident in parenterally fed patients, as risk for any complication increased by a factor of 1.58 for each 1 mmol/L glycemic increment above 6.3 mmol/L.^10^ Of note, the use of nutrition support in our study was frequent with more than half of the population being prescribed enteral and/or parenteral nutrition. Insulin reinforces the anabolic effect of nutrition support while preventing hyperglycemia-related oxidative damage and risk of infection.^24,34–36^ In this context, the benefits of tight glucose control may depend on the use of concomitant nutrition therapy, as supported by a recent multicentre randomized controlled trial which did not demonstrate improved patient outcomes with tight glucose versus more liberal glucose control in the absence of parenteral nutrition in critical care patients.^37^ However, despite being referred to as liberal, mean daily glucose levels in the respective group were still lower than those of our control group [8.0 (7.1; 9.2) vs 8.6 (7.0; 10.6) mmol/L].

Differences in glucose control were accompanied by a significantly lower TDD in the UC group. This suggests a reluctance to treat hyperglycemia by the hospital care team, especially among patients who are insulin naïve,^38^ despite prior evidence showing that correction of hyperglycemia is associated with improved outcomes among hospitalized patients, independent of diabetes status.^15,39^ Clinical inertia (ie, failure to initiate insulin therapy) observed in the UC group may be the result of insufficient awareness of hyperglycemia, perhaps exacerbated by the impact of hospital staff shortage and heavy workload of personnel with conflicting priorities.

Findings from previous studies support the notion that patients without insulin therapy before admission benefit from adequate glycemic control in hospitals and might require even more stringent goals than patients with diabetes. In a retrospective cohort study of over 7000 surgical patients, the hyperglycemia-associated risk for postoperative complications was up to 2-fold higher in patients without diabetes compared with the equivalent hyperglycemic levels in patients with a history of diabetes.^40^ This evidence supports the recommended glucose target of <7.8 mmol/L in non-diabetes inpatients, as advised by the consensus statement of the American Diabetes Association.^41^ Insulin delivery, if performed safely, not only corrects hyperglycemia but also lowers inflammation and promotes an anabolic state needed for postoperative recovery, providing multifaceted treatment advantages.^18,42^ Of note, the use of an adaptive FCL system allows to control glucose levels on the basis of individualized targets and to achieve tighter control without increasing the risk of hypoglycemia, opening new avenues to study individualized therapy regimens, tailored to comorbidity levels and preadmission glycemia. The benefits of individualized blood glucose targets based on preadmission glucose control (using HbA1c) are supported by observational studies in critical care patients, but could not be substantiated in a recent randomized controlled trial due to a higher rate of hypoglycemic events with lower targets.^43–45^

We have previously demonstrated the superiority of the FCL approach compared with UC in a mixed elective surgery population requiring insulin for perioperative glucose control.^28^ In comparison with a previous study, the current trial demonstrated even greater benefit of FCL conducted in 2 tertiary hospital centres and in a more metabolically challenging population (majority undergoing pancreatic surgery, more patients requiring postoperative nutrition support and longer surgery duration as well as hospital stay). Considering the FCL approach as a means to save time for clinical staff (FCL was shown to reduce the time required for perioperative glucose management 2.1–4.5 times compared with standard insulin therapy^46^), may endorse its use for the management of frail and comorbid patients with complex medical needs and in settings with low staffing levels. The satisfactory performance of an FCL system depends on the accuracy of CGM, which may interfere with medications (eg, acetaminophen, vitamin C) and abnormal biochemistry.^47^ In the present study we used the Dexcom G6 technology, which demonstrated acceptable performance across a heterogeneous population including critical care and noncritical patients.^48^ During abdominal surgery and the postoperative period, the Dexcom G6, when compared against arterial glucose values, showed satisfactory performance with mean absolute relative differences ranging from 9.4% to 12.7%.^49,50^ However, intraoperative use of interstitial CGM may preclude certain surgical settings such as cardiac surgery with extracorporeal circulation, where a mean absolute relative difference of 23.8% was found.^51^

We acknowledge some limitations of the present study. The sample size was small and powered to evaluate glucose control but not postoperative outcome data, which usually requires several hundreds of subjects.^52^ The small sample size and the large proportion of patients undergoing pancreatic surgery limit the generalizability of the present study to other patients undergoing major abdominal surgery. In particular, our study did not include cardiac surgery patients, in whom good glycemic control led to lower morbidity (eg, less surgical site infections) and mortality.^53^ In addition, study devices were primarily managed by the study team with ward nurse involvement for only defined tasks, and the integration with the clinical information system was lacking. Therefore, it is unclear whether FCL glucose management can seamlessly integrate into the clinical daily routine. Glucose management in the UC group lacked process standardization, which may have led to poorer glucose control but is challenging to implement.

## CONCLUSIONS

FCL glucose control proved to be an effective and safe approach to attenuate hyperglycemia and glucose fluctuations in major abdominal surgery patients with high metabolic stress exposure. Future studies with appropriate sample sizes are needed to consolidate its integration in clinical workflows as well as its potential to improve clinical outcomes such as nutrition status and recovery after surgery.

## Supplementary Material
