# Supplementary material for: Perioperative Fully Closed-loop Versus Usual Care Glucose Management in Adults Undergoing Major Abdominal Surgery: A Two-centre Randomized Controlled Trial
Source: Ann Surg. 2024 Sep 30;281(5):732–40. doi: 10.1097/SLA.0000000000006549 (PMC11974617; doi:10.1097/SLA.0000000000006549)
Supplement: Supplementary file 1 [file sla-281-732-s001.docx]

| **Table S1: Inclusion and exclusion criteria** |
| --- |
|  |
| **Inclusion criteria:** |
| - Age 18 years or over |
| - Pre-existing or anticipated (surgery-induced) diabetes other than type 1 diabetes |
| - Expected to require insulin treatment in the perioperative period |
| - Planned for elective major abdominal surgery at the University Hospital Bern or Basel expected to last ≥ 90 minutes, defined as colorectal, pancreatic, gastric (except bariatric surgery) and hepatic (≥2 segments) surgery |
|  |
| **Exclusion criteria:** |
| - Physical or psychological condition likely to interfere with the normal conduct of the study and interpretation of the study results as judged by the investigator |
| - Likely discharge earlier than 72 hours |
| - Known or suspected allergy to insulin used in this clinical trial |
| - Type 1 diabetes |
| - Pregnancy, planned pregnancy, or breast feeding |
| - Lack of safe contraception for female participants of childbearing potential for the entire study duration |
| - Medically documented allergic/irritative skin reactions towards dressings/adhesives (glue) of plasters |
| - Serious skin diseases located at places of the body, which potentially are possible to be used for localisation of the glucose sensor |
| - Illicit drug abuse or prescription drug abuse |
| - Incapacity to give informed consent |
| - Not willing to wear study devices 24/7 |

**Table S2: CONSORT 2010 checklist**


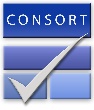
CONSORT 2010 checklist of information to include when reporting a randomised trial*

| Section/Topic | Item No | Checklist item | Reported on page No |
| --- | --- | --- | --- |
| Title and abstract | | | |
|  | 1a | Identification as a randomised trial in the title | 1 |
|  | 1b | Structured summary of trial design, methods, results, and conclusions (for specific guidance see CONSORT for abstracts) | 2-3 |
| Introduction | | | |
| Background and objectives | 2a | Scientific background and explanation of rationale | 3-4 |
|  | 2b | Specific objectives or hypotheses | 4 |
| Methods | | | |
| Trial design | 3a | Description of trial design (such as parallel, factorial) including allocation ratio | 5 |
|  | 3b | Important changes to methods after trial commencement (such as eligibility criteria), with reasons | NA |
| Participants | 4a | Eligibility criteria for participants | 5 |
|  | 4b | Settings and locations where the data were collected | 6 |
| Interventions | 5 | The interventions for each group with sufficient details to allow replication, including how and when they were actually administered | 6-7 |
| Outcomes | 6a | Completely defined pre-specified primary and secondary outcome measures, including how and when they were assessed | 8 |
|  | 6b | Any changes to trial outcomes after the trial commenced, with reasons | NA |
| Sample size | 7a | How sample size was determined | 9 |
|  | 7b | When applicable, explanation of any interim analyses and stopping guidelines | NA |
| Randomisation: |  |  |  |
| Sequence generation | 8a | Method used to generate the random allocation sequence | 5 |
|  | 8b | Type of randomisation; details of any restriction (such as blocking and block size) | 5-6 |
| Allocation concealment mechanism | 9 | Mechanism used to implement the random allocation sequence (such as sequentially numbered containers), describing any steps taken to conceal the sequence until interventions were assigned | 6 |
| Implementation | 10 | Who generated the random allocation sequence, who enrolled participants, and who assigned participants to interventions | 6 |
| Blinding | 11a | If done, who was blinded after assignment to interventions (for example, participants, care providers, those assessing outcomes) and how | 6 |
|  | 11b | If relevant, description of the similarity of interventions | NA |
| Statistical methods | 12a | Statistical methods used to compare groups for primary and secondary outcomes | 9 |
|  | 12b | Methods for additional analyses, such as subgroup analyses and adjusted analyses | NA |
| Results | | | |
| Participant flow (a diagram is strongly recommended) | 13a | For each group, the numbers of participants who were randomly assigned, received intended treatment, and were analysed for the primary outcome | 10, Figure S1 |
|  | 13b | For each group, losses and exclusions after randomisation, together with reasons | 10 |
| Recruitment | 14a | Dates defining the periods of recruitment and follow-up | 10 |
|  | 14b | Why the trial ended or was stopped | NA |
| Baseline data | 15 | A table showing baseline demographic and clinical characteristics for each group | 10, Table 1 |
| Numbers analysed | 16 | For each group, number of participants (denominator) included in each analysis and whether the analysis was by original assigned groups | 10 |
| Outcomes and estimation | 17a | For each primary and secondary outcome, results for each group, and the estimated effect size and its precision (such as 95% confidence interval) | 10-11 |
|  | 17b | For binary outcomes, presentation of both absolute and relative effect sizes is recommended | NA |
| Ancillary analyses | 18 | Results of any other analyses performed, including subgroup analyses and adjusted analyses, distinguishing pre-specified from exploratory | NA |
| Harms | 19 | All important harms or unintended effects in each group (for specific guidance see CONSORT for harms) | 12 |
| Discussion | | | |
| Limitations | 20 | Trial limitations, addressing sources of potential bias, imprecision, and, if relevant, multiplicity of analyses | 15 |
| Generalisability | 21 | Generalisability (external validity, applicability) of the trial findings | 15 |
| Interpretation | 22 | Interpretation consistent with results, balancing benefits and harms, and considering other relevant evidence | 13-16 |
| Other information | | |  |
| Registration | 23 | Registration number and name of trial registry | 5 |
| Protocol | 24 | Where the full trial protocol can be accessed, if available | NA |
| Funding | 25 | Sources of funding and other support (such as supply of drugs), role of funders | 1 |

| **Table S3: Clavien-Dindo classification of surgical complications** | | |
| --- | --- | --- |
|  | **FCL = 18** | **UC = 19** |
| Clavien-Dindo Index^a^ |  |  |
| Grade 0 | 3 (16.7%) | 6 (31.6 %) |
| Grade I | 4 (22.2%) | 9 (50.0%) |
| Grade II | 24 (133.3%) | 17 (89.5%) |
| Grade III | 6 (33.3%) | 4 (21.1%) |
| Grade IV | 6 (33.3%) | 0 |
| Grade V | 2 (11.1%) | 0 |

Data are n (%). FCL, fully closed-loop; UC, usual care.

^a^ One subject may be attributed to more than one grade, except for grade 0.

| **Table S4: Pre-admission and post-discharge CGM metrics** | | |
| --- | --- | --- |
| **Pre-admission** | **FCL = 15** | **UC = 14** |
| Proportion of time spent at glucose concentration |  |  |
| 3.9-10.0mmol/L (%) | 53 ± 39.9 | 54.4 ± 32.3 |
| >10.0 mmol/L (%) | 30.4 [13.1; 94.9] | 51.4 [19.4; 66.0] |
| >20.0 mmol/L (%) | 0.0 [0.0; 3.7] | 0.0 [0.0; 0.7] |
| <3.9 mmol/L (%) | 0.0 [0.0; 0.0] | 0.0 [0.0; 0.0] |
| <3.0 mmol/L (%) | 0.0 [0.0; 0.0] | 0.0 [0.0; 0.0] |
| Mean glucose concentration (mmol/L) | 11.5 ± 4.5 | 10.4 ± 3.0 |
| SD glucose concentration (mmol/L) | 2.2 ± 1.1 | 2.5 ± 1.1 |
| CV of glucose concentration (%) | 20.2 ± 8.0 | 23.0 ± 6.8 |
| No. of days with CGM data | 3.1 ± 2.2 | 3.8 ± 1.8 |
|  |  |  |
| **Post-discharge** | **FCL = 15** | **UC = 17** |
| Proportion of time spent at glucose concentration |  |  |
| 3.9-10.0mmol/L (%) | 66.2 ± 19.4 | 69.1 ± 28.3 |
| >10.0 mmol/L (%) | 32.4 [17.4; 44.2] | 26.1 [4.5; 42.8] |
| >20.0 mmol/L (%) | 0.0 [0.0; 1.2] | 0.0 [0.0; 0.0] |
| <3.9 mmol/L (%) | 0.1 [0.0; 0.5] | 0.0 [0.0; 0.3] |
| <3.0 mmol/L (%) | 0.0 [0.0; 0.1] | 0.0 [0.0; 0.0] |
| Mean glucose concentration (mmol/L) | 9.2 ± 1.5 | 9.3 ± 2.2 |
| SD glucose concentration (mmol/L) | 2.6 ± 1.2 | 2.1 ± 1.1 |
| CV of glucose concentration (%) | 28.1 ± 11.4 | 22.0 ± 7.7 |
| No. of days with CGM data | 12.7 ± 5.7 | 13.8 ± 5.4 |

Data are mean±SD or median [25th; 75th percentile]. FCL, fully closed-loop; UC, usual care; CGM, continuous glucose monitoring.


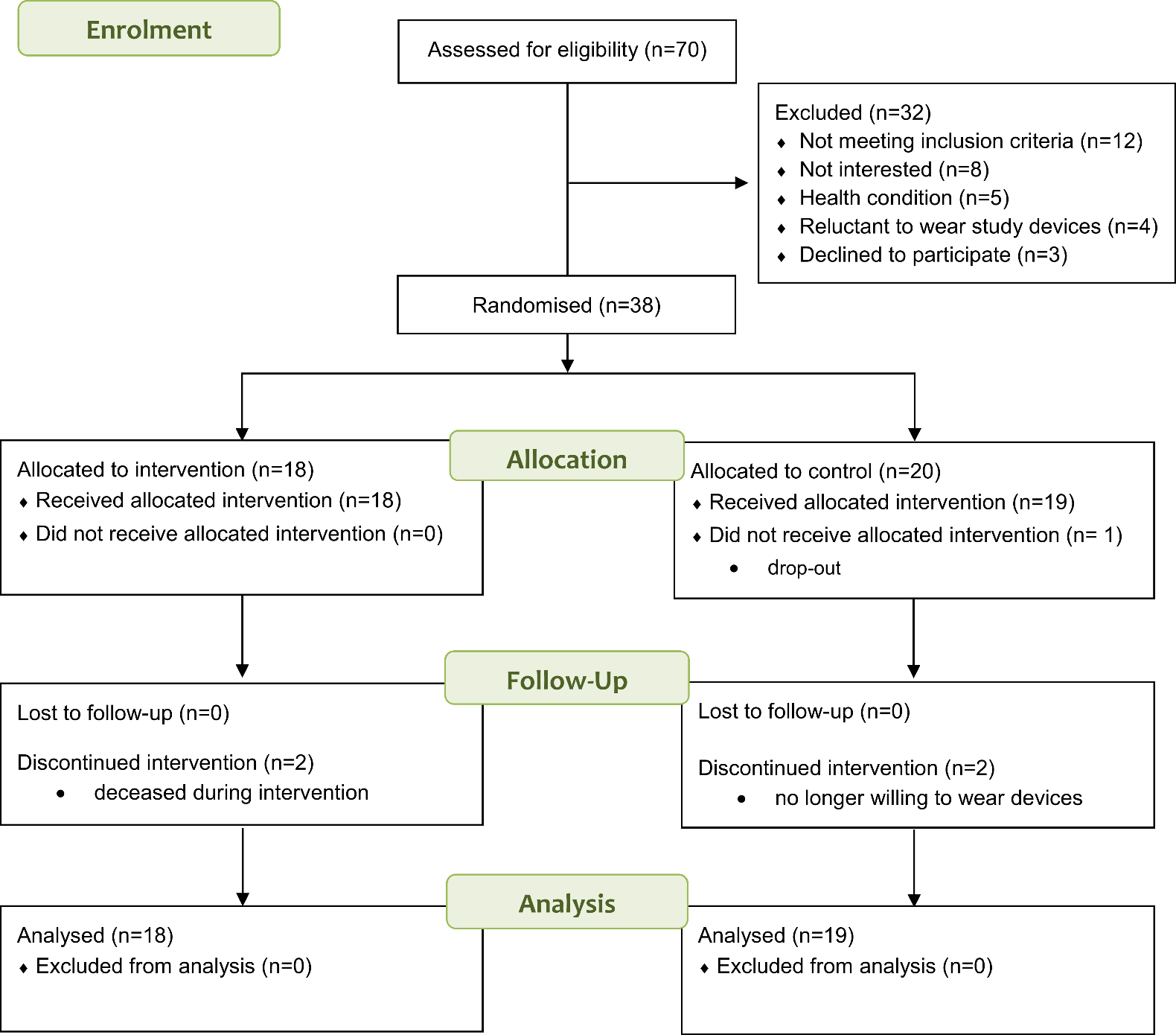
**Figure S1: Consort Flow Diagram**


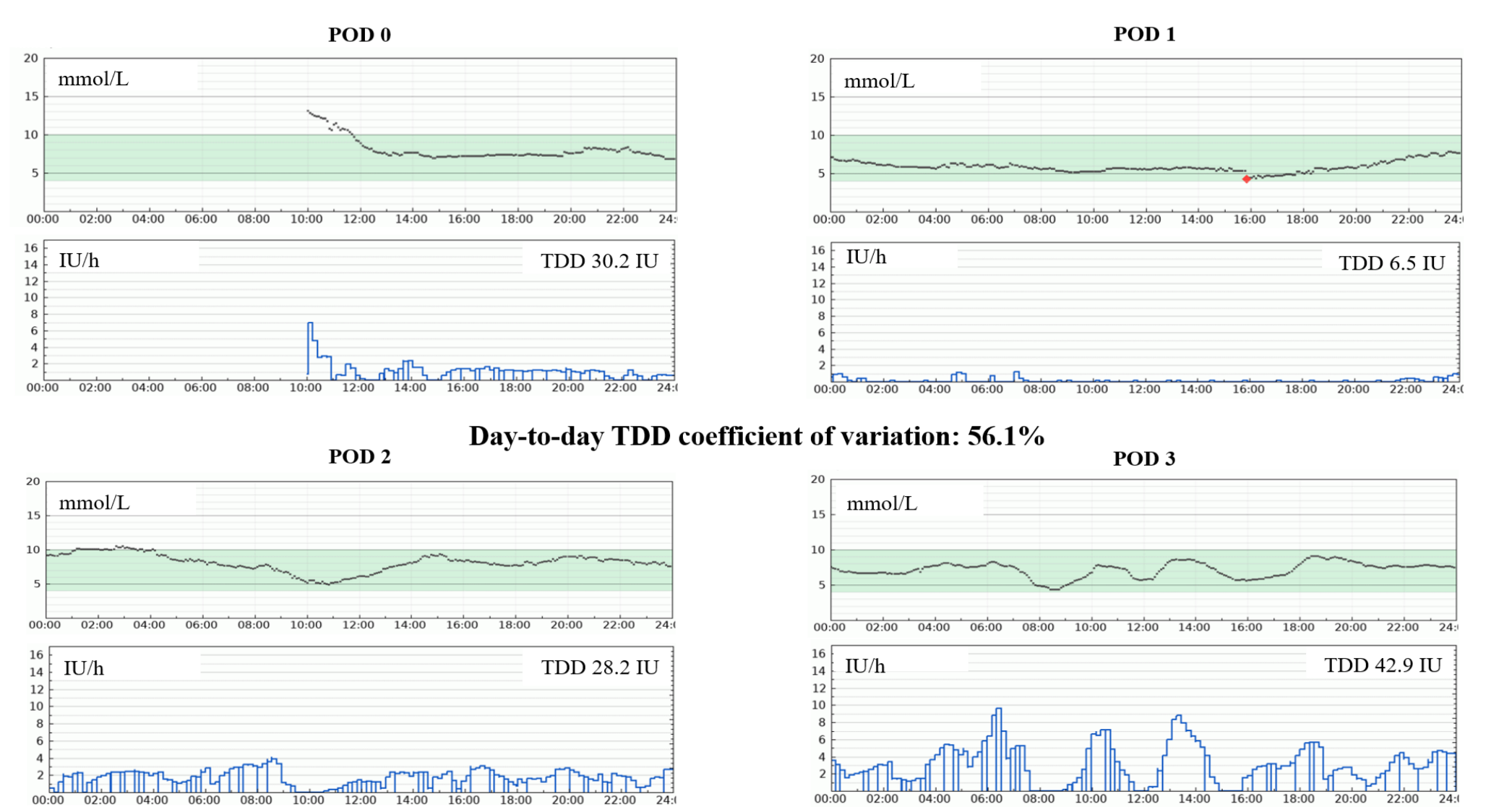
**Figure S2: Time course of exogenous insulin requirements in a patient undergoing total pancreatectomy**

Example of the fully closed-loop insulin delivery profile in a patient undergoing total pancreatectomy, from time of surgery until postoperative day 3. Y axis shows insulin delivery rate (units/hour) and sensor glucose concentration (mmol/L), X axis displays time of day. Coefficient of variation of insulin requirements is calculated for TDD between four days displayed.

*IU, international units; POD, postoperative day; TDD, total daily insulin dose.*
